# A Manual of Anatomy

**Published:** 1896-08

**Authors:** 


A Manual of Anatomy. By Irving S. Haynes, Ph. B., M. D., Adjunct Professor and Demonstrator of Anatomy in the Medical Department of the New York University ; Visiting Surgeon to the Harlem Hospital, etc. 8vo, pp. 680, with 130 half-tone illustrations and 42 diagrams. Price, $2.50 net. Philadelphia : W. B. Saunders, 925 Walnut street. 1896.

This volume does not aim to be a complete treatise upon anatomy. The author deals principally with the viscera and their relations to the surface of the body. Only so much of the anatomy of the extremities has been given as seemed required. The bones, joints and minuter parts requiring special preparation for dissection have been intentionally omitted. The descriptions of the various viscera are terse but complete, and in the case of the more important organs include the essential points in the history of their development.

The chapter upon the peritoneum is excellent and so clearly written as to surely help the student in better understanding it.

The index is exceptionally complete and accurate. In short, the book contains a large amount of accurate and useful information in comparatively small compass.

We wish we could speak as enthusiastically about the illustrations. Why authors and publishers will deface their books with illustrations that mystify rather than elucidate we cannot understand. Until photography is so far advanced that it can represent tissues faithfully, not only in form but color, it should not be made use of in text-books. The photographic illustrations in this book are many of them little more than blots and convey little or no information to one familiar with anatomy. It may well be asked, therefore, of how much real service they can be to those beginning the study of this science, the very ones for whom this manual is intended.

The type and general make-up of this volume are excellent and do great credit to the publishers. J. P.

